# Spaceflight induces novel regulatory responses in Arabidopsis seedling as revealed by combined proteomic and transcriptomic analyses

**DOI:** 10.1186/s12870-020-02392-6

**Published:** 2020-05-27

**Authors:** Colin P. S. Kruse, Alexander D. Meyers, Proma Basu, Sarahann Hutchinson, Darron R. Luesse, Sarah E. Wyatt

**Affiliations:** 1 Department of Environmental and Plant Biology, Ohio University, Athens, OH USA; 2 Interdisciplinary Molecular and Cellular Biology Program, Ohio University, Athens, OH USA; 3 Department of Biological Sciences, Southern Illinois University Edwardsville, Edwardsville, IL USA

**Keywords:** Gravity, Spaceflight, Arabidopsis, Gravitropism, Transcriptomics, Proteomics, Phosphorylation, Post-transcriptional gene regulation, Plastid

## Abstract

**Background:**

Understanding of gravity sensing and response is critical to long-term human habitation in space and can provide new advantages for terrestrial agriculture. To this end, the altered gene expression profile induced by microgravity has been repeatedly queried by microarray and RNA-seq experiments to understand gravitropism. However, the quantification of altered protein abundance in space has been minimally investigated.

**Results:**

Proteomic (iTRAQ-labelled LC-MS/MS) and transcriptomic (RNA-seq) analyses simultaneously quantified protein and transcript differential expression of three-day old, etiolated *Arabidopsis thaliana* seedlings grown aboard the International Space Station along with their ground control counterparts. Protein extracts were fractionated to isolate soluble and membrane proteins and analyzed to detect differentially phosphorylated peptides. In total, 968 RNAs, 107 soluble proteins, and 103 membrane proteins were identified as differentially expressed. In addition, the proteomic analyses identified 16 differential phosphorylation events. Proteomic data delivered novel insights and simultaneously provided new context to previously made observations of gene expression in microgravity. There is a sweeping shift in post-transcriptional mechanisms of gene regulation including RNA-decapping protein DCP5, the splicing factors GRP7 and GRP8, and AGO4,. These data also indicate AHA2 and FERONIA as well as CESA1 and SHOU4 as central to the cell wall adaptations seen in spaceflight. Patterns of tubulin-α 1, 3,4 and 6 phosphorylation further reveal an interaction of microtubule and redox homeostasis that mirrors osmotic response signaling elements. The absence of gravity also results in a seemingly wasteful dysregulation of plastid gene transcription.

**Conclusions:**

The datasets gathered from Arabidopsis seedlings exposed to microgravity revealed marked impacts on post-transcriptional regulation, cell wall synthesis, redox/microtubule dynamics, and plastid gene transcription. The impact of post-transcriptional regulatory alterations represents an unstudied element of the plant microgravity response with the potential to significantly impact plant growth efficiency and beyond. What’s more, addressing the effects of microgravity on AHA2, CESA1, and alpha tubulins has the potential to enhance cytoskeletal organization and cell wall composition, thereby enhancing biomass production and growth in microgravity. Finally, understanding and manipulating the dysregulation of plastid gene transcription has further potential to address the goal of enhancing plant growth in the stressful conditions of microgravity.

## Background

Plants use external stimuli to adapt to their environment. The most consistent of these stimuli is gravity, the sensing of which allows a plant to properly orient its growth. Since the first plants were sent to space nearly 50 years ago on the NASA Skylab, researchers have utilized the microgravity environment of low Earth orbit to study plant growth, development, and response. Examining plants in microgravity offers insight into the mechanisms of gravity signaling under terrestrial conditions and helps to forge the way toward use of plant cultivation in long-term human space habitation.

Early microgravity research examined plant growth and architecture on the whole-plant level. Coupling spaceflight experiments with molecular techniques provides the hope of uncovering some of the elusive molecular pathways driving the gravitropic response. Although costly and complex, new technology has made molecular spaceflight research feasible. Benefits from such studies are two-fold: 1) understanding how plants respond to microgravity may offer insight into optimizing their growth and success in cultivation for beyond-earth food and bioregenerative life support systems, and 2) the possibility of uncovering cryptic signaling components in the gravity response by utilizing the disruption in the gravity stimulus to track molecular changes that reveal the mechanisms behind gravity-related plant growth on Earth. Within the last decade, a handful of spaceflight experiments have produced transcriptomic and proteomic data. Microarray [1–7], RNA-seq [8], and protein mass spec [9–12] experiments have provided snapshots of the molecular environment under microgravity conditions. Because of the low cost and ubiquitous nature of next generation sequencing technology, RNA-seq has become the go-to molecular method for comparing gene expression between multiple samples. It can produce a high-resolution snapshot of gene activation at a given time. However, this approach embraces the implicit assumption that changes to transcript levels correspond to changes in protein abundance. RNA-seq also cannot reveal protein turnover or post-translational modifications.

Combining high-resolution transcriptomic techniques such as RNA-seq with proteomics provides a more comprehensive, robust approach to understanding the molecular approach an organism adopts to a new environment than either technique individually. Data presented here were gathered by RNA-seq and protein mass spectrometry providing, for the first time, simultaneous information on transcript abundance, peptide abundance, and changes in post translational modifications (PTMs) in plants grown in the absence of gravity. To gain a more complete view of the molecular environment in microgravity, differentially abundant soluble and membrane proteins were identified via tandem mass spectroscopy with supporting RNA-seq analysis of transcript abundance. Additionally, we were able to track changes in phosphorylation and other PTMs of proteins.

Data presented here suggest that the changes induced by microgravity cannot be adequately described by gene expression alone. Analysis of these samples shows that RNA-seq, protein MS, and PTM each tell their own story, contributing intriguing clues to the effects of microgravity at the cellular level. These investigations reveal global perturbations across multiple levels of regulation, novel gravity-responsive PTM events, and a more complete picture of how plants respond to microgravity conditions.

## Results

The BRIC-20 experiment examined the proteomes and transcriptomes of etiolated 3-day old Arabidopsis seedlings grown aboard the International Space Station (ISS) and identical samples grown on Earth. Arabidopsis seeds were densely planted on 0.5x MS agar plates supplemented with sucrose and germinated on orbit. After 76 h of etiolated growth within the Biological Research in Cannisters (BRIC) hardware, seedlings were fixed with RNAlater and frozen until return to earth. Total protein, divided into soluble and insoluble fractions, and RNA, were extracted from the samples. RNA-seq reads were obtained by Illumina HiSeq 2500. Proteins were ITRAQ-labeled, and discovery proteomics was performed using LC-MS/MS. Total protein levels, as well as PTMs, were compared between flight and ground samples with a decision rule of *P* ≤ 0.1 to allow for PTM detection in the absence of phosphoenrichment. MS/MS data revealed a subset of phosphorylation events that were significantly (*P* ≤ 0.1) differentially abundant in space compared to earth.

**Fig. 1:**
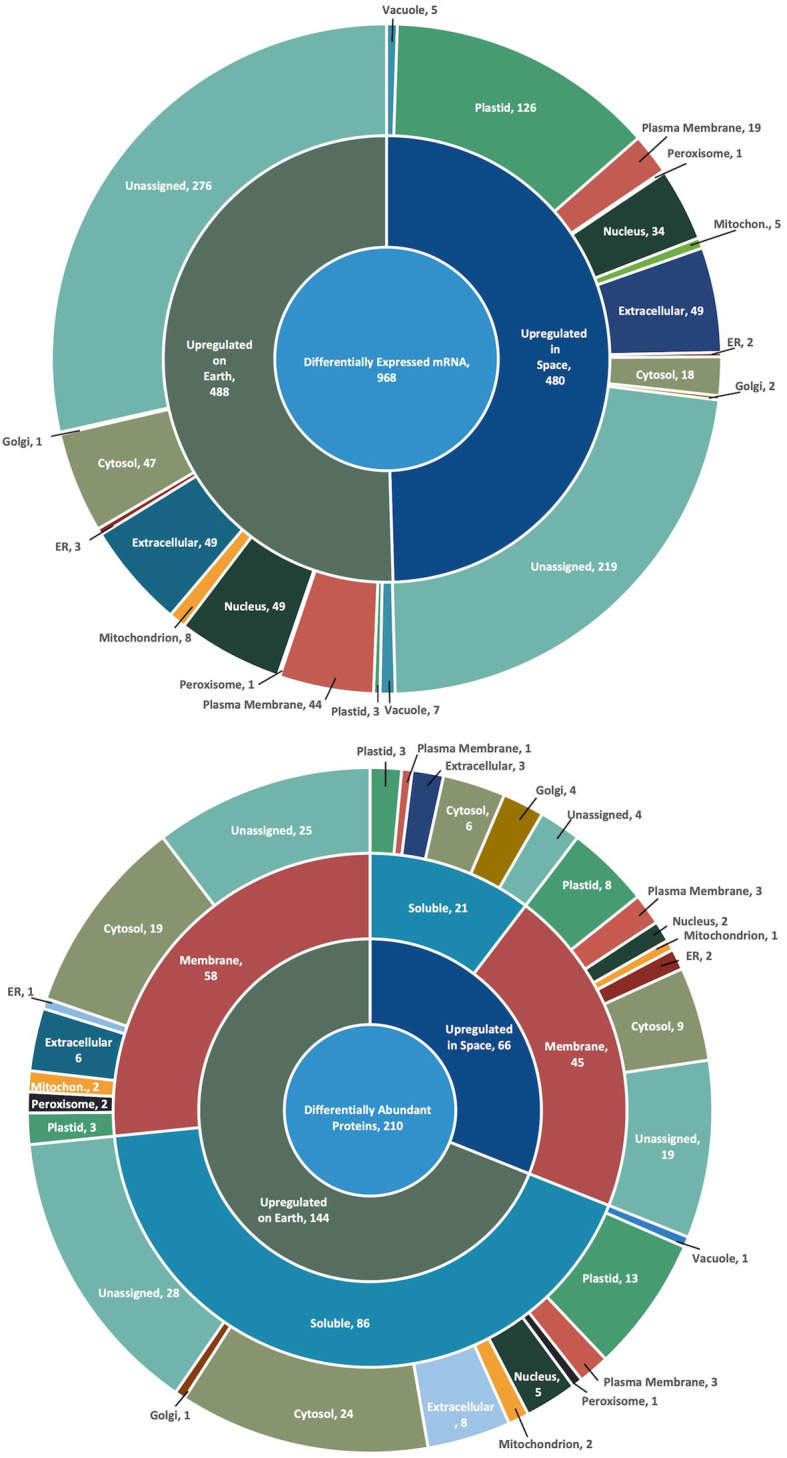
Overview of transcripts (top) and proteins (bottom) differentially expressed during spaceflight. RNA sequencing and protein mass spectrometry was performed on space-flown Arabidopsis seedlings and compared to ground controls. The number adjacent to each sector label indicates the count of genes/proteins that occupy the slice. Subcellular localizations are shown in the outermost circles. SUBA4 [13] (V.4.6.0, accessed June 2017) was used to identify the subcellular localizations. Gene/protein accessions and expression values are provided in Supplemental File 1

Overall, 968 genes, 107 soluble proteins, and 103 membrane proteins were identified as significantly differentially expressed (*P* ≤ 0.05) between spaceflight and ground conditions (Fig. 1). At the transcript level, 480 genes were upregulated and 488 genes were downregulated (at a L_2_FC of ≥ ±1; 2-fold differential expression) in space as compared to on Earth; 45 membrane and 21 soluble proteins were more abundant in space and 58 membrane and 86 soluble proteins were less abundant in space (L_2_FC of ≥ ± 0.2). In addition to the differentially expressed proteins, we also observed 16 peptides that were differentially phosphorylated in microgravity (*P* ≤ 0.1).

The subcellular localization of the differential proteins and the proteins encoded by the differentially expressed transcripts was assessed using SUBA4 [13]. One of the most overt differences in cellular localization was the > 25% of transcripts upregulated in spaceflight being localized to the plastid compared to < 1% of the transcripts upregulated on Earth (Fig. 1). This change was not reflected in the protein data and provides a primary indication of dysregulation of plastid genes in the absence of gravity despite, or perhaps because of, the lack of chloroplast development in etiolated seedlings. Extracellular localization was only observed in the soluble proteins downregulated in space, with 8 extracellular localized proteins. These proteins are primarily regulators of oxidation and cell wall dynamics (Table 1).

**Table 1:** Extracellular localized soluble proteins downregulated by spaceflight

**Locus Identifier**	**Gene Description**	**Primary Gene Symbol**
AT2G04690	Pyridoxamine 5-phosphate oxidase family protein	–
AT3G28200	Peroxidase superfamily protein	–
AT5G63180	Pectin lyase-like superfamily protein	–
AT5G56870	Beta-galactosidase 4	BETA-GALACTOSIDASE 4 (BGAL4)
AT4G34180	Cyclase-family protein that is a negative regulator of cell death that regulates pathogen-induced symptom development.	CYCLASE1 (CYCLASE1)
AT4G08950	Phosphate-responsive 1 family protein	EXORDIUM (EXO)
AT1G14540	Peroxidase superfamily protein	PEROXIDASE 4 (PER4)
AT5G59090	Subtilase 4.12	SUBTILASE 4.12 (SBT4.12)

### Gene ontology (GO) analysis reveals processes altered in microgravity

**Fig. 2:**
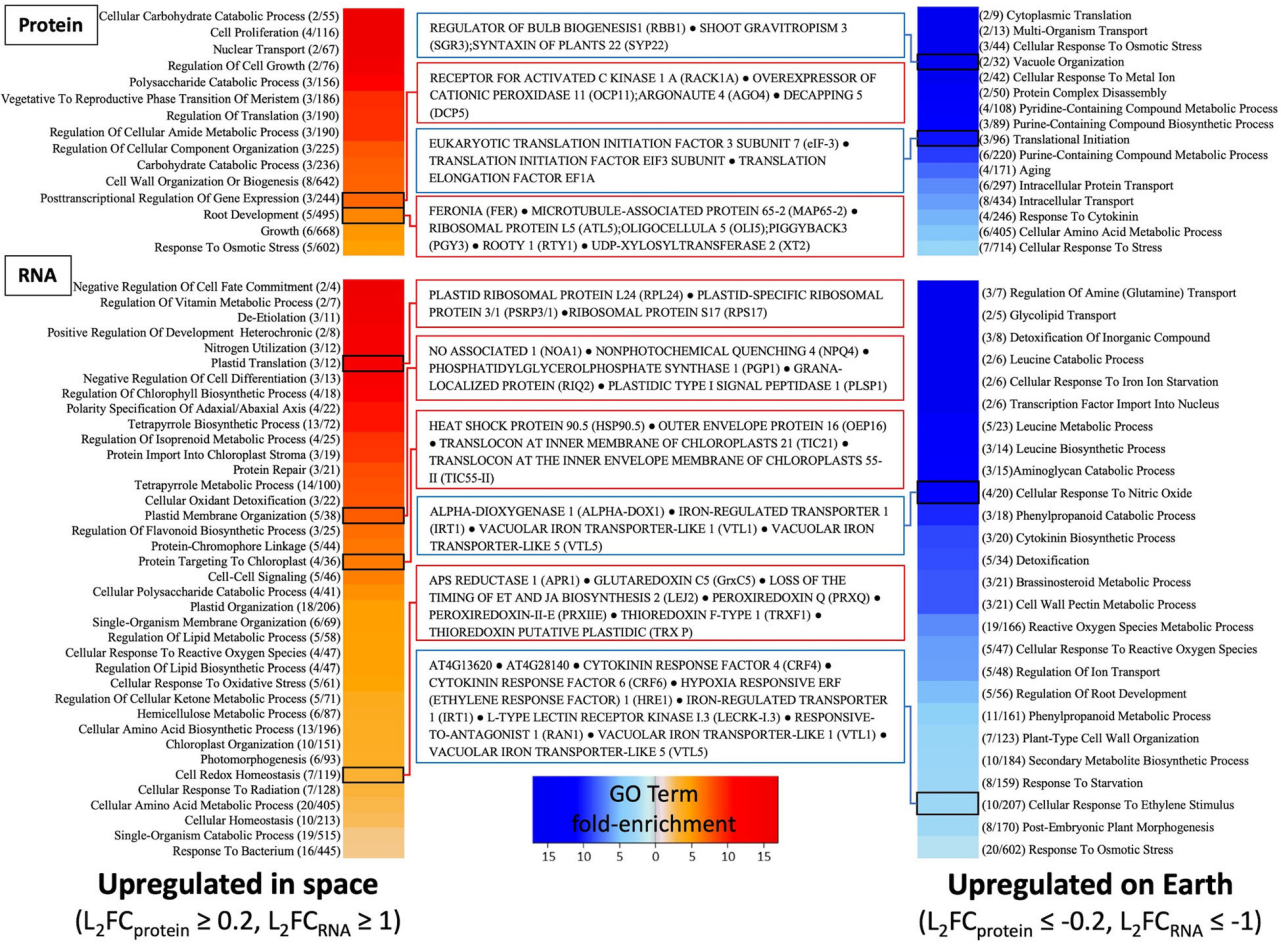
Heat map of a gene ontology enrichment analysis of genes/proteins differentially upregulated in space (left) or upregulated on Earth (right). The central callouts list the differentially expressed genes/proteins within the selected GO term. Enrichment was performed for GO terms for biological processes using Orange (V.3.7.0, GO terms were accessed July 2017). Only transcripts with a log-fold change ±1 (FDR ≤ 0.05) or proteins with a log-fold change ±0.2 (*P* ≤ 0.05) were included in the analysis. The upregulated and downregulated genes were run separately, and the resultant GO enrichments were thinned to include GO terms with a *p*-value ≤0.05 using the Bonferroni correction for multiple comparisons. Numbers next to the terms represent the number of genes or proteins that appeared in that term, followed by the total number genes or proteins associated with that term. Supporting data can be found in Supplemental File 2

To determine the global physiological effects of spaceflight conditions, an enrichment analysis of GO terms was performed. In an effort to identify trends in altered physiological function, four discrete groupings of molecular data were analyzed: 1) proteins that were in more abundance in spaceflight samples; 2) proteins more abundant on Earth, 3) transcripts more abundant in the spaceflight samples, and 4) transcripts more abundant on Earth (Fig. 2). Four key features of transcriptomic and proteomic datasets reveal novel physiological adaptations in spaceflight and simultaneously demonstrate the utility of this mixed ‘omics approach. First, genes regulating cell wall organization processes are enriched among upregulated proteins and downregulated transcripts. The differential phosphorylation, protein, and gene expression data all reveal specific aspects of a nuanced regulatory shift in cell-wall related processes as a part of the change associated with microgravity. Second, redox homeostasis and a broad set of GO terms related to reactive oxygen species production and metabolism are enriched among the up and down regulated transcripts but not mirrored in the differentially expressed proteins. A third intriguing feature is the upregulation of plastid-localized gene transcription despite the seedlings being etiolated (as the hardware allows no light to enter). Finally, there is a pervasive shift in the regulation of translation and post-transcriptional regulation as a whole that is solely enriched at the protein level.

### Post-transcriptional gene regulation, alternative splicing, and post-translational modifications

**Fig. 3:**
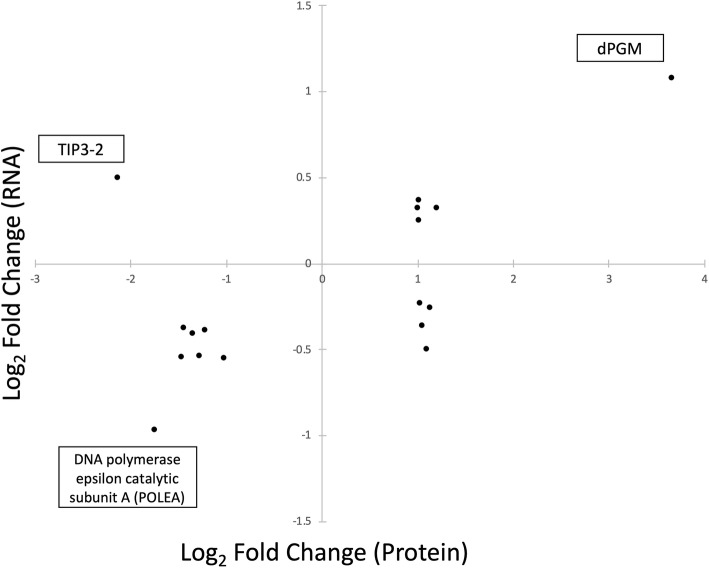
Genes/proteins differentially expressed in both transcript and protein datasets. Of the 17 genes, 12 have coordinated expression patterns (i.e. increased or decreased in both the transcript and protein data), while 5 genes exhibit opposing expression patterns between datasets. X-axis = transcript expression, Y-axis = protein expression. Boxes indicated gene/protein name of the associated point. Some of the most extreme expression patterns highlight TONOPLAST INTRINSIC PROTEIN 3–2 (AT1G17810), a cofactor-dependent phosphoglycerate mutase (dPGM, AT5G04120), and a DNA-polymerase epsilon catalytic subunit A (AT1G19530) within the datasets

To determine if any of the proteins identified were altered independent of transcript levels, we compared the lists of significantly altered transcripts (*P* ≤ 0.05; L_2_FC of ≥1) and the list of significantly altered proteins (*P* ≤ 0.05; L_2_FC of ≥0.2). Of the genes and proteins showing differential expression/abundance as a result of spaceflight, only 17 appeared in both the RNA and protein datasets (Fig. 3, Table 2) representing 2 and 8% of mRNAs and proteins, respectively. Of the 17 in this group, 12 showed consistent direction of expression (i.e. both increased or both decreased). The remaining five showed expression in opposing directions between the transcript and protein. Hypergeometric distribution analysis indicated a significant (*p* = 0.036) correlation between these gene sets when considering only the genes that show consistent direction of expression, and a more dramatic correlation when including all 17 overlapping genes (*p* = 9.7 e^− 4^). A cofactor-dependent phosphoglycerate mutase (dPGM, AT5G04120) predicted to be involved in glycolysis was upregulated in both datasets in response to spaceflight [14]. Given the etiolated seedlings were grown on sucrose media, this shift in a regulatory component of glycolysis may be representative of an important metabolic target for spaceflight adaptation. Inversely, AT1G19530—an epsilon catalytic subunit A of DNA polymerase—was consistently downregulated in proteomic and transcriptomic analyses (Fig. 3). Notably, a probable aquaporin protein belonging to the tonoplast intrinsic protein family (TIP3.2) was significantly more abundant in space despite TIP3.2 transcripts being more than four-fold downregulated. Of the 17 genes and proteins coordinately differentially expressed, nearly 30% of these genes show opposite directions of differential expression like TIP3.2. Thus, post-transcriptional gene regulation may have important implications for adjustment to spaceflight.

**Table 2:** Genes altered in response to spaceflight by protein and RNA quantification

**TAIR ID**	**Symbol**	**Membrane *p*-value**	**Membrane** **L**_**2**_**FC**	**Soluble** ***p*-value**	**Soluble L**_**2**_**FC**	**Gene L**_**2**_**FC**	**Gene FDR**
AT5G04120	dPGM (unofficial)	0.000	1.080	NA	NA	3.663	0.000
AT1G17810	TIP3–2	0.050	0.500	NA	NA	−2.131	0.020
AT5G19370	–	0.000	0.370	NA	NA	1.011	0.003
AT4G18480	CHLI1	0.043	0.320	NA	NA	1.207	0.000
AT3G16290	FTSHI2	0.023	0.320	NA	NA	1.003	0.005
AT4G19710	AK-HSDH II	0.032	0.250	NA	NA	1.020	0.001
AT1G11580	PMEPCRA	0.026	−0.390	NA	NA	−1.223	0.005
AT3G48730	GSA2	0.001	−0.500	NA	NA	1.102	0.000
AT1G66280	BGLU22	0.001	−0.550	NA	NA	−1.024	0.003
AT1G19530	POLEA (unofficial)	0.001	−0.970	NA	NA	−1.746	0.000
AT5G08280	HEMC	NA	NA	0.014	−0.232	1.030	0.008
AT3G52960	PRXIIE	NA	NA	0.047	−0.257	1.139	0.001
AT5G09650	PPa6	NA	NA	0.045	−0.359	1.057	0.001
AT5G59090	SBT4.12	NA	NA	0.042	−0.377	−1.443	0.000
AT5G43780	APS4	NA	NA	0.048	−0.410	−1.351	0.000
AT5G56870	BGAL4	NA	NA	0.032	−0.539	−1.280	0.000
AT3G11930	–	NA	NA	0.002	−0.544	−1.467	0.000

To determine if post-transcriptional regulation is responsible for the low correlation between transcriptomic and proteomic data sets, we filtered the differentially expressed RNAs and proteins to identify genes in post-transcriptional regulation. Transcript and protein levels for many ribosomal subunits were altered between Earth and microgravity, which may have an impact on the translational dynamics of the cell. Twelve transcripts for ribosomal proteins were differentially expressed, with only one, a structural component, downregulated nearly 8-fold in space (Table 3). The remaining 11 were upregulated in the spaceflight samples. Medina [15] suggested that changes in ribosome biosynthesis as a result of microgravity caused an uncoupling of cellular growth and cellular proliferation [15]. The differential expression of various tRNAs and the increased abundance of proteins related to post-transcriptional regulation of gene expression and translational initiation (enriched in proteins upregulated and downregulated in spaceflight, respectively) suggest an even more substantial impact on regulatory dynamics induced by spaceflight (Fig. 2; Supplemental File 2). The increased protein expression of RNA DECAPPING PROTEIN 5 (DCP5, AT1G26110, L_2_FC_membrane_ = 0.32) and the RNA-silencing protein ARGONAUTE 4 (AGO4, AT2G27040, L_2_FC_soluble_ = 0.44, L_2_FC_RNA_ = 0.78) further indicate an increased mRNA turnover and siRNA-mediated gene silencing while coping with microgravity. Perhaps the most novel finding relating to this regulatory shift is found in the PTM data. EUKARYOTIC TRANSLATION INITIATION FACTOR ISOFORM 4G-1, GTP BINDING ELONGATION FACTOR TU FAMILY, ELONGATION FACTOR 1-DELTA 1, and a Trigger Factor Type Chaperone family protein all had differential levels of phosphorylation (Table 4) suggesting an additional mechanism for regulating translation. Among these phosphorylation events, we also see evidence for differential splicing as an adaptation to spaceflight. The RNA-binding proteins GRP7 and GRP8, both regulators of alternative splicing [16], were preferentially phosphorylated on Earth at the S118 residue in response to microgravity (L_2_FC_phospho_ = − 0.39 and − 0.54, respectively) (Table 5). This suggests an alteration in splicing patterns within the plant in response to spaceflight. A splice aware analysis of transcripts identified 24 genes with at least two transcripts differentially expressed (Fig. S1). VILLIN 1, PEROXIN 11E, NATURAL RESISTANCE-ASSOCIATED MACROPHAGE PROTEIN 1 isoforms that are inversely differentially expressed by spaceflight. For example, the actin binding protein VILLIN 1 isoforms AT2G29890.2 (L_2_FC_RNA_ > 5) and AT2G29890.3 (L_2_FC_RNA_ < − 7.5) approach complete silencing in space and on earth respectively (Additional file 3: Fig. S1). The altered ratio of gene isoforms confirms that transcripts are subject to altered processing in microgravity.

**Table 3:** Genes with ontology tags relating to post-transcriptional regulation that were altered in response to spaceflight

**TAIR ID**	**Description**	**L**_**2**_**FC**	**FDR**
AT4G14250	Structural constituent of ribosome	−2.66	0.001
AT3G13120	30S ribosomal protein S10, chloroplastic	1.00	0.015
AT2G33450	50S ribosomal protein L28, chloroplastic	1.01	0.021
AT5G30510	Ribosomal protein S1	1.01	0.002
AT5G54600	50S ribosomal protein L24, chloroplastic	1.01	0.012
AT1G79850	30S ribosomal protein S17, chloroplastic	1.02	0.002
AT5G20180	Ribosomal protein L36	1.03	0.019
AT5G40950	50S ribosomal protein L27, chloroplastic	1.05	0.002
AT2G24090	Ribosomal protein L35	1.06	0.001
AT4G11175	Translation initiation factor IF-1, chloroplastic	1.08	0.002
AT3G27160	Ribosomal protein S21 family protein	1.11	0.001
AT1G68590	30S ribosomal protein 3–1, chloroplastic	1.13	0.025
AT1G32990	50S ribosomal protein L11, chloroplastic	1.18	0.003

**Table 4:** Proteins with ontology tags relating to post-transcriptional regulation that were altered in response to spaceflight

**TAIR ID**	**Description**	**L**_**2**_**FC**
**Protein**		
AT1G66070	Translation initiation factor eIF3 subunit	−0.94
AT4G24820	26S proteasome non-ATPase regulatory subunit 6 homolog	−0.76
AT3G62870	60S ribosomal protein L7a-2	−0.66
ATCG00830	50S ribosomal protein L2, chloroplastic	−0.65
AT1G18070	Translation elongation factor EF1A/initiation factor IF2gamma family protein	−0.52
AT5G44320	Eukaryotic translation initiation factor 3 subunit 7 (eIF-3)	−0.47
AT4G02930	GTP binding elongation factor Tu family	−0.42
AT5G43960	Nuclear transport factor 2 (NTF2) family protein with RNA binding domain	−0.40
AT3G27830	50S ribosomal protein L12–1, chloroplastic	−0.35
AT1G09830	Phosphoribosylamine--glycine ligase, chloroplastic	−0.33
AT4G29040	26S proteasome regulatory subunit 4 homolog A	−0.32
AT3G53020	60S ribosomal protein L24–2	−0.32
AT3G53970	Probable proteasome inhibitor	−0.23
AT1G73990	Serine protease SPPA, chloroplastic	0.21
AT5G39740	60S ribosomal protein L5–2	0.24
AT3G25520	60S ribosomal protein L5–1	0.25
AT1G26110	Protein decapping 5	0.32
AT5G13650	elongation factor family protein	0.35
AT2G27040	Protein argonaute 4	0.44
AT1G02560	ATP-dependent Clp protease proteolytic subunit 5, chloroplastic	0.56
AT1G78880	Ubiquitin-specific protease family C19-related protein	0.81
**Phosphoproteome**		
AT5G57870	Eukaryotic translation initiation factor isoform 4G-1	−0.47
AT4G02930	GTP binding elongation factor Tu family	0.44
AT1G30230	Elongation factor 1-delta 1	0.45
AT5G55220	Trigger factor type chaperone family protein	0.65

**Table 5:** Alterations to the phosphoproteome in response to spaceflight

**TAIR ID**	**Description**	**Subcellular Location**	**Peptide Sequence** **(Phosphorylation site in red)**	**L**_**2**_**FC**
AT4G02930	GTP binding elongation factor	mitochondrion	NMITGAAQMDGGILVVSGPDGPMPQTK	0.48
AT3G01570	Oleosin family protein	vacuole	THSHQLQVHPQR	0.37
AT4G32410	Cellulose synthase 1	plasma membrane, Golgi	TTSGPLGPSDR	0.23
AT4G37870	PEP carboxykinase1	cytosol	SAPTTPINQNAAAAFAAVSEEER	−0.04
AT1G64740	Tubulin alpha-1	cytosol	TIQFVDWCPTGFK	−0.24
AT1G04820	Tubulin alpha-4	plasma membrane	TIQFVDWCPTGFK	−0.24
AT4G14960	Tubulin alpha-6	cytosol	TIQFVDWCPTGFK	−0.24
AT5G19770	Tubulin alpha-3	cytosol	TVQFVDWCPTGFK	−0.26
AT4G30190	AHA2	plasma membrane	GLDIETPSHYTV	−0.36
AT2G21660	AtGRP7	nucleus	SGGGGGYSGGGGSYGGGGGR	−0.39
AT5G20650	Copper transporter 5	vacuole	SSSGVSAPLIPK	−0.44
AT5G57870	Translation initiation factor	cytosol, nucleus	GESVVSNLVPVQSASR	−0.46
AT4G39260	AtGRP8	nucleus	SGGGGGYSGGGGGGYSGGGGGGYER	−0.54
AT5G55220	Chaperone family protein	plastid	EVENSISEFK	−0.61
AT2G30930	Unknown protein	plasma membrane	ATSALSEAK	−0.87
AT4G27450	Aluminum induced protein	cytosol	VDSEGVLCGANFK	−1.23

In addition to regulators of translation and RNA splicing/degradation, components of the ubiquitin ligase protein degradation pathway were also identified in the spaceflight samples. Three E3 ligase mRNAs were altered by a L_2_FC of ≥ ±1 including ARABIDOPSIS TOXICOS EN LEVADURA 9 (ATL9, AT2G35000, L_2_FC_RNA_ = − 1.1), BOI-RELATED GENE 2 (BRG2, AT1G79110, L_2_FC_RNA_ = 1.9), and RING-H2 FINGER PROTEIN 2B (RHA2B, AT2G01150, L_2_FC_RNA_ = 1.0). ATL9, expressed in an NADPH-dependent matter, has been implicated in pathogen response due to its chitin-mediated expression, but is not upregulated in response to the canonical defense hormones [17]. BRG2 is a member of a subclass of RING-type E3 ligases which contribute *Botrytis cinerea* resistance and mediate stress and pathogen responses [18]. Finally, the E3 ligase RHA2B has an additive effect with its closest homolog, RHA2A, in regulating ABA and drought response [19]. In addition to the standard E3 ligases, five members of the F-box superfamily, PHLOEM PROTEIN 2-A13 (PP2-A13, AT3G61060, L_2_FC_RNA_ = − 2.0), AT3G27150 (L_2_FC_RNA_ = − 1.7), PHLOEM PROTEIN 2-A14 (PP2-A14, AT5G52120, L_2_FC_RNA_ = 1.5), and AT1G23390 (L_2_FC_RNA_ = − 1.3) were differentially expressed.

Other components of the regulated proteolysis pathway were also altered. REGULATORY PARTICLE AAA-ATPASE 2A (RPT2a, L_2_FC_Soluble_ = − 0.32) and a 26S proteasome regulatory subunit (RPN7, AT4G24820, L_2_FC_Soluble_ = − 0.76) were both downregulated in spaceflight samples. NUCLEAR ENCODED CLP PROTEASE 5 (CLPP5, L_2_FC_Soluble_ = 0.56), SIGNAL PEPTIDE PEPTIDASE (SPPA, L_2_FC_Membrane_ = 0.21), and the peptidase SHOU4 (AT1G78880, L_2_FC_Membrane_ = 0.81) were all upregulated in space. An additional 23 proteases were altered at the transcript level (Additional file 1). Overall, microgravity-grown plants appear to display changes in the capacity to produce, regulate, and degrade proteins.

### Cell Wall synthesis in the spaceflight environment

Microgravity has been shown to have a significant impact on plant growth [20, 21]. In these datasets, the most obviously impact is seen in differential expression and regulation of components of cell wall modification. At the protein level, a number of UDP-D-Xylose synthases, transferases, and hydrolases (XT2, GUT1, XS1,XS2, XTH19) were upregulated in microgravity, along with a putative plasmodesmata localized BETA-1,3-GLUCANASE (BG_PPAP, AT5G42100).

Quantitative tracking of PTMs has revealed several novel aspects of spaceflight acclimation in plants, including alterations in cell wall synthesis (Table 5). PLASMA MEMBRANE PROTON ATPASE 2 (AHA2) was differentially phosphorylated between flight and ground controls. AHA2 has been implicated as the link between auxin and acid growth. The redistribution of auxin in response to tropic stimulus initiates transcription of SAUR19, which in turn inhibits a PP2C-D phosphatase allowing for AHA2-mediated acidification of the cell wall and subsequent cellular elongation [22]. The phosphorylated form of AHA2 was more abundant on Earth (L_2_FC_phospho_ = − 0.36) (Table 5). Components involved in cellulose deposition also showed altered transcription. CELLULOSE SYNTHASE 1(CESA1) phosphorylation was increased in spaceflight samples (L_2_FC_phospho_ = 0.23) (Table 3) while, Tubulin-α 1, 3,4 and 6, required for cellulose patterning, were preferentially phosphorylated on Earth (L_2_FC_phospho_ = − 0.24, − 0.26, − 0.24 and − 0.24 respectively) (Table 5) at T349; phosphorylation at T349 is known to encourage microtubule depolymerization [23].

While many plant processes are modulated by auxin, auxin-mediated asymmetric cellular elongation is the primary effector of directional growth responses, including gravitropism, and is an essential component in the regulation of cell wall growth and elongation. Many of the auxin-related pathways are clearly perturbed in response to microgravity, with representatives from many aspects of auxin signaling and response appearing in either the protein or the gene expression dataset. Both of the enzymes in the primary synthesis pathway from tryptophan to IAA are represented. The first, L-tryptophan-pyruvate-aminotransferase 1 (L_2_FC_RNA_ = 0.82), is upregulated nearly two-fold. The transcript for the second enzyme, indole-3-pyruvate-monooxegenase (YUCCA6, L_2_FC_RNA_ = -0.86), was downregulated nearly two-fold in spaceflight samples. This expression pattern could result in an increased level of Indole-3-pyruvate —the final precursor in auxin synthesis—along with a decrease in conversion of Indole-3-pyruvate to IAA (primary active form of auxin) because of the decreased level of YUCCA6. Several of the genes responsible for auxin redistribution were also present in the datasets. Five auxin carriers were differentially expressed in total: PIN2 (AT5G57090, L_2_FC_RNA_ = -0.77), PIN-like7 (AT5G65980, L_2_FC_RNA_ = -0.95), and ABCB15 (AT3G28345, L_2_FC_RNA_ = -0.87) were down-regulated in space, while PIN-FORMED 4 (PIN4, AT2G01420, L_2_FC_RNA_ = 0.77) and LAX2 (AT2G21050, L_2_FC_RNA_ = 0.87) were up-regulated, suggesting that space-grown plants are altering their auxin distribution in response to the space-flight environment. In addition to auxin biosynthesis and transport, auxin perception and response were also perturbed. AUXIN SIGNALING F BOX PROTEIN 1 (AFB1, AT4G03190, L_2_FC_membrane_ = − 0.11), a member of the TIR1 family, was less abundant in microgravity in the protein dataset. Auxin-responsive proteins IAA5 (L_2_FC_RNA_ = 2.83) and IAA28 (L_2_FC_RNA_ = -0.84) genes were found to be differentially expressed, as well as Auxin-response factors ARF4 (L_2_FC_RNA_ = 0.81) and ARF11 (L_2_FC_RNA_ = 0.72). Two small auxin-upregulated RNA (SAUR) genes (both downregulated) and five SAUR-like genes (3 downregulate, 2 upregulated) were differentially expressed in microgravity. These have been shown to be auxin-responsive regulators of adaptive growth, and may be responsible for modulating new growth patterns in the absence of gravity [24]. Whether auxin is the predominant regulator of cell wall related adaptions remains to be seen, however, the characterization provided by transcript, protein and PTM data provides an enhanced understanding of cell wall regulatory dynamics—a primary target for optimization of plant growth in space.

### Influences on plastid function and regulation

Genes and terms relating to plastid function were among the most pronounced features of the subcellular localization (Fig. 1) and the gene ontology enrichment analysis (Fig. 2). Notably, the enrichment of genes associated with chlorophyll biosynthetic process, protein import into chloroplast stroma, plastid membrane organization, protein targeting to chloroplast, and chloroplast organization were seen in the transcript dataset (Fig. 2; Additional file 2). These results were unexpected because seedlings were sealed inside PDFU’s, which were then sealed inside the BRIC. This environment provides no light to initiate chlorophyll synthesis and chloroplast development Although plastids play an important role in gravity perception through the sedimentation of dense amyloplasts in the root cap and shoot endodermis, many of the differentially expressed genes (e.g. chlorophyll synthase) relate to photosynthesis, rather than amyloplast-related functions. This may suggest a microgravity-induced disruption in plastid operation that goes beyond gravity perception and may impact metabolic-based plastid processes.

Furthermore, gene ontology analysis for the transcript data revealed significant enrichment in terms such as plastid translation, protein import into the stroma, targeting to chloroplast, and plastid organization (Fig. 2; Supplemental File 2). The increased transcript expression of plastid ribosomal proteins SVR8 (AT5G54600, L_2_FC_RNA_ = 1.01), PSRP3/1 (AT1G68590, L_2_FC_RNA_ = 1.13), and RPS17 (AT1G79850, L_2_FC_RNA_ = 1.02), translocons TIC55-II (AT2G24820, L_2_FC_RNA_ = 1.01), and TIC21 (AT2G15290, L_2_FC_RNA_ = 1.03), as well as OUTER ENVELOPE PROTEIN 16 (OEP16) (AT2G28900, L_2_FC_RNA_ = 1.57), PLASTIDIC TYPE I SIGNAL PEPTIDASE 1 (PLSP1) (AT3G24590, L_2_FC_RNA_ = 1.04), and GRANA-LOCALIZED PROTEIN (RIQ2) (AT1G74730, L_2_FC_RNA_ = 1.22) may indicate a disruption in plastid physiology in response to microgravity. The transcript-specific alterations in biological functions and localizationsuggests alterations in plastid homeostasis. This phenomenon may, in part, be linked to the reduced negative impact on growth of PhyD-deficient mutants in the spaceflight environment [25]. Identifying the regulatory mechanism responsible for these potentially “wasted” transcripts may provide an ideal target for engineering plants that remain hearty in microgravity.

### Altered redox state and ROS signaling

Reactive oxygen species have emerged as important components of plant signaling, but may also be produced as byproducts of stress metabolism [26]. Transcripts for L-ascorbate oxidase (AT5G21100, L_2_FC_RNA_ = 1.43), L-ascorbate peroxidase S (AT4G08390, L_2_FC_RNA_ = 0.96), monodehydroascorbate reductase (AT1G63940, L_2_FC_RNA_ = 0.98), as well as ascorbate transporters (AT4G00370, L_2_FC_RNA_ = 0.84; AT2G27810, L_2_FC_RNA_ = 0.68; AT2G34190, L_2_FC_RNA_ = 0.82) were all up-regulated in the spaceflight samples. In addition to ascorbate related genes, respiratory burst oxidase homolog (RBOH) genes were altered. RBOHB (AT1G09090, L_2_FC_RNA_ = − 1.38) and RBOHI (AT4G11230, L_2_FC_RNA_ = − 1.03) (superoxide-producing NADPH oxidases) were down regulated in microgravity. RBOH genes are known mediators of reactive oxygen species (ROS) signaling [26, 27].

To understand the physiological impact of the altered expression of ROS regulators, we examined the oxidation state of the proteome from our LC-MS/MS results. The insoluble spaceflight samples showed a prevalence of oxidized proteins and decreased oxidation in the soluble samples. Analysis of the membrane protein fraction identified a total of 3659 oxidized peptides, with 174 peptides showing significantly (*P* ≤ 0.05) altered prevalence between space flown and ground control seedlings. Of those significantly altered, 79% showed a greater prevalence of the oxidized form in space. The trend of increased oxidation may be due in part to the decreased level of several extracellular peroxidase proteins in microgravity. This trend was reversed in the soluble proteins with 88% of the 265 significantly altered peptides showing decreased oxidation in the space-flown seedlings.

## Discussion

### Multiple ‘omics approaches yield distinct but comparable results

**Fig. 4:**
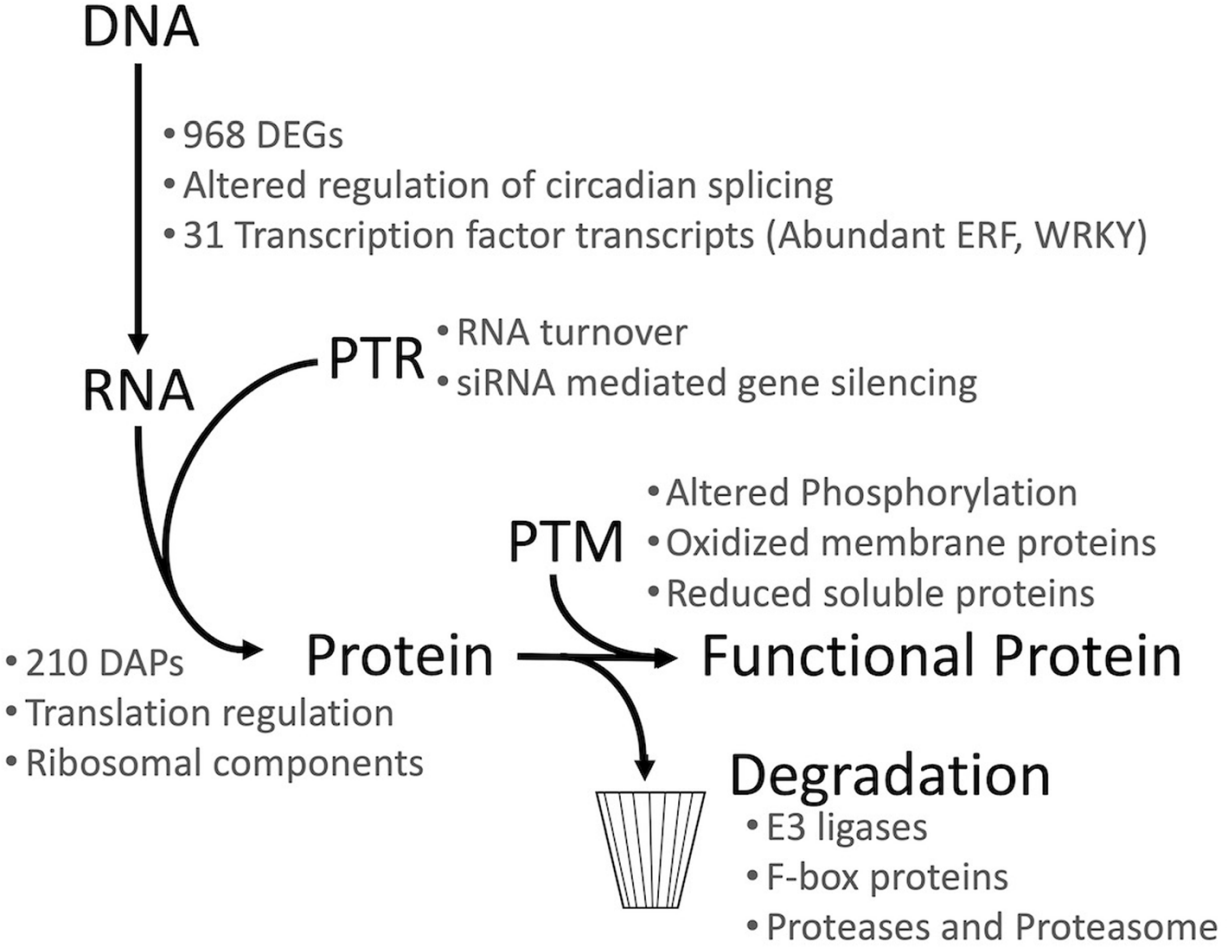
Summary of the results and insight provided from the transcript (RNA), protein, and post-translational modification (PTM) datasets. Transcript expression profiles were characterized by RNA-seq and protein expression profiles were characterized and quantified by iTRAQ LC-MS/MS. Expression and fragmentation data characterized the regulatory dynamics induced by adaptation to the spaceflight environment in *Arabidopsis thaliana*. Gene Ontology analysis identified PTR, PTM, and Degradation candidate genes. DEGs = differentially expressed genes, DAPs = differentially abundant proteins, PTR = post-transcriptional regulation

The transcript, protein, and post-translational modification data reported here demonstrate that cellular perturbations caused by microgravity are likely pervasive across all “levels” of regulation. The evidence within these datasets shows that transcription, translation, PTM, and protein turnover are all altered by microgravity (Fig. 4). This global dysregulation suggests that much of the molecular landscape is affected in spaceflight conditions.

One striking (but not entirely unexpected) feature of these data is the low correlation between differentially expressed transcripts and differentially abundant proteins (Fig. 3). As the central dogma of biology is most generally reduced to the statement of “DNA to RNA to protein”, the impacts of regulatory mechanisms between transcript and peptide are often forgotten. The resultant differences in the representative mRNA and proteins can be profound, and lack of a correlative relationships, comparable to observations in this experiment, are also observed in a number of studies across model species [28–31]. This misalignment of transcript and peptide data is not a discrepancy, but rather it provides a more comprehensive view of the molecular and regulatory landscape under the chosen treatment conditions.

### Dysregulation of plastid transcripts

Plants grown in microgravity face a variety of challenges including altered gas exchange and the absence of a gravity vector. Plastids play a central role in a plant’s ability to sense and respond to gravity, but also function to store resources collect light energy, and generate cellular signaling components. In this context, it is unsurprising to see a wide variety of plastid-localized and plastid-produced genes differentially regulated in the flight samples (Fig. 1, Fig. 2). However, the presence of several chloroplast-related proteins, specifically those related to photosynthesis, was unexpected. It is not immediately clear how these genes are functioning in this case, as the growth conditions inside the BRIC hardware prevent all light from reaching the plants. It is possible that the stresses of space initiate signaling pathways that induce low levels of chlorophyll production. However, visually the plants appeared to be etiolated (data not shown) so any upregulation of chlorophyll production is small. As these genes appear in the transcriptomic but not the proteomic data set, their expression may indicate a wasteful effort at enhancing light signaling to compensate for the absence of gravity. The absence of any classical directional cue may remove a regulatory feedback that halts the expression of the core plastid components thus resulting in continuous wasteful expression. In either case, understanding the cause of this wasted transcriptional activity could be a ready target for the production of plants optimized for spaceflight applications. This dysregulation may be involved in the improvement to spaceflight adaptation seen in mutants lacking the photosynthetic regulatory component PhyD [25].

### Global alterations to redox homeostasis

Our results also show alterations in genes and proteins that regulate ROS. Although potentially involved in stress response signaling, they can also be damaging byproducts of stress-induced metabolic signaling [32]. In space, plants responded by increasing the levels of several ROS quenchers such as L-ascorbate oxidase, L-ascorbate peroxidase, and monodehydroascorbate reductase. They also limited production or superoxide-producing RBOH genes. As an overall profile, this makes sense. Microgravity is inherently a stressful environment. Limiting the production of harmful stress byproducts is likely important for plants to cope. Despite this, the data show the activation of ATL9 (a NADH-oxidase dependent expression event) indicating an increased response to ROS. The increased oxidation of the membrane proteome despite the reduced expression of extracellular peroxidases (Table 1) in contrast to the reduced state of the soluble proteome indicates that there exists an increased oxidative extracellular environment and a reduced oxidative potential of the cytosol. ROS has a significant role in modulating the cytoskeletal dynamics of actin [33–35] and microtubules [36]. Thus, stabilization of microtubules in microgravity via decreased phosphorylation of the α-tubulin/β-tubulin interface site of four α-tubulin may represent a ROS-related alteration to cytoskeletal dynamics [23]. This modification and microtubule depolymerization is induced rapidly upon hyperosmotic stress [23]. As with previous spaceflight experiments, response to osmotic stress is enriched among differentially expressed genes, likely due to the absence of the structural guide provided by the constant pull of gravity. Increased microtubule stability has the potential to enhance cytoskeletal structure in the absence of gravity. ROS-induced microtubule dynamics and microtubule-mediated ROS attenuation as seen in hyperosmotic stress responses thus provides a mechanism for a specific compensatory adaptation to space. As microtubules provide the patterning for CESA-mediated cellulose deposition, enhancing the impact of this compensatory adaptation (i.e. microtubule stabilization) could be a highly effective target for enhancing plant growth and biomass generation in the absence of gravity.

### Cell Wall dynamics and Auxin on the ISS

While many plant processes are modulated by auxin, auxin-mediated cellular elongation is the primary effector of differential growth and organ re-orientation [37]. In the absence of gravity (the primary guide of etiolated seedling orientation and growth direction), the plant may default to secondary guiding signals such as touch or micro-gradients in water or nutrients. In this case, the upregulation of PIN, LAX and ABC transporters may represent the plant quickly and transiently responding to many smaller gradients that are no longer overshadowed by gravity. However, auxin is involved in a myriad of cellular processes, so it remains possible that the alterations in auxin-related genes are a byproduct of some other aspect of physiology and not related to gravity or tropisms.

The ability of proteomic sequencing to reveal post-translational modifications is a major benefit of the approach. In the case of gravity signaling, the traditional approaches of genetic mutant screens and RNA-seq have failed to identify some of the signaling components of the pathway, suggesting that post-translational modification of potentially redundant proteins, rather than alterations of their levels, may be responsible. We identified post-translational phosphorylation of auxin signaling components. Plasma Membrane Proton H+ ATPase 2 (AHA2) acidifies the cell wall downstream of auxin redistribution [37]. We found the C-terminal domain of AHA-2 to be more phosphorylated in the Earth samples as compared to the space-flown seedlings indicating a reduced activity. Inhibition of AHA2 is also post-translationally regulated in FERONIA-dependent manner [37] which is simultaneously increased in abundance in space doubly indicating a suppression of AHA2 and acid growth of the cell wall.

As the plant cell wall evolved in the presence of earth’s gravity, the question of if and how microgravity influences cell wall content has been of interest to researchers for some time. Arabidopsis hypocotyls have lower levels of cellulose when grown in microgravity [20, 21]. However, no cellulose synthase genes (CESA) were observed in the BRIC20 differential transcript or protein datasets (though one CES-like gene was observed in the RNA data). CESA1 was phosphorylated more often in response to microgravity (Table 5). CESA1 is required for primary cell wall biosynthesis, and phosphorylation of CESA proteins negatively modulates their synthetic efficiency [38]. As with AHA2, we observed multiple mechanisms for the inhibition of CESA by the nearly two-fold enhancement of soluble protein SHOU4 [39], which suppresses cellulose synthesis by the regulation of CESA trafficking to the plasma membrane [40].

## Conclusions

The unified transcriptomic and proteomic characterization of space-flown *Arabidopsis thaliana* demonstrate the value in employing various ‘omics approaches for data acquisition. The regulatory landscape of the cell is difficult to decipher with transcript data alone, such that the addition of proteomic data provides a more complete picture of plant response to spaceflight and microgravity. Ultimately, we have characterized a significant impact of the spaceflight environment on post-transcriptional regulation, redox homeostasis, plastid gene transcription, and cell wall synthesis. While some of these responses to microgravity have been previously studied or observed, the transcriptomic and proteomic studies unified to produce the most comprehensive understanding of these events to date and provide new direction and strategies for plant engineering. The role of CESA1 and AHA2 along with their potential regulation by SHOU4 and FERONIA, respectively, provide novel insights into the decreased cellulose deposition previously observed in spaceflight [20, 21]. In addition, the interface of redox homeostasis and microtubule dynamics provides a relevant physiological adaptation to the spaceflight environment that may result in altered oxidative stress while also enhancing the structural integrity cells in the absence of gravity. Altered regulation of specific elements of cell wall synthesis and microtubule stability, along with the production of plastid transcripts, provide three new targets to engineer plants for optimal growth during long-term spaceflight missions. The sweeping alterations to mRNA turnover, silencing, and splicing, as well as initiation/elongation of protein translation, protein turnover, and other aspects of post-transcriptional regulation may raise more questions than answers and necessitates further study. This regulatory shift indicates that questions regarding spaceflight physiology will not be adequately addressed by transcriptomic studies. Finally, we note that phosphoenriched proteomics remain an unmet need to characterize plant response to microgravity and will provide essential insights into the regulatory mechanisms of plant adaptation to space.

## Methods

### Plant material

*Arabidopsis thaliana* (L.) Heyn. var. Columbia (Arabidopsis Biological Resource Center, Stock ID: CS22625), were used for all experiments. Aliquots of 0.016 ± 0.002 g of seeds (approximately 800–900 seeds per plate) were surface sterilized with 30% (v/v) bleach and 0.1% (v/v) Tween-20 for 2 min, washed with sterile water (5x), then dried onto 60 mm round filter paper to facilitate plating. Seeds were spread onto 60 mm round Petri plates containing 0.5 X Murashige and Skoog (MS) media (Caisson, Smithfield, UT) with 1% sucrose and 1% agar, cold stratified overnight, and provided a 1 h white light treatment to facilitate consistent germination.

### Spaceflight parameters

Twenty-two seeded plates were then individually integrated into Petri Dish Fixation Units (PDFUs), and RNALater (Ambion, Carlsbad, CA) was loaded into the liquid reservoir. PDFUs were then tested for leaks and loaded into Biological Research in Canister (BRIC) hardware for flight: five plates plus a HOBO data logger in two BRICs and six plates in the remaining two BRICs. All BRICs were put into cold stowage (4 °C) prior to launch. The experiment was launched aboard Space-X 5 on January 10, 2015. Upon docking of the Dragon capsule to the International Space Station (ISS), the BRICs were removed from cold stowage and placed at ambient temperature (ca. 21 °C). After 76 h, the PDFUs were actuated releasing RNAlater into the Petri dish for seedling fixation. Samples were incubated at ambient temperature in RNAlater for 12 h then placed at − 80 °C in the MELFI freezer aboard ISS. Twenty-two additional plates were integrated into hardware as ground controls and maintained under identical growth conditions and processing on a 48 h delay. These were held at Kennedy Space Center with environmental conditions aboard ISS provided via downlink. After the spaceflight samples returned from ISS, both spaceflight and ground control plates were removed from the hardware while frozen and packed in dry ice at Kennedy Space Center, then transported to Ohio University in a temperature-controlled vehicle. In addition to the Kennedy Ground control, two additional controls were set up to check if the spaceflight hardware or the preservative RNAlater were causing any significant changes in the transcriptome and proteome of Arabidopsis seedlings. Details of the hardware and preservative studies have been published in Basu et al. [41] and Kruse et al. [42], respectively. All pertinent datasets are publicly available on the NASA GeneLab repository.

### RNA extraction and processing

RNA was extracted according to Kruse et al. [39, 41]. In brief, three biological replicates, one plate per replicate, were used for RNA-seq analysis. RNA was extracted using a RNeasy Plant Mini Extraction Kit (Qiagen) according to standard protocols. RNA integrity (RIN ≥ 8) was verified using an Agilent 2100 Bioanalyzer. RNA samples were sent to the Genome Technology Access Center in the Department of Genetics at Washington University School of Medicine, St Louis, MO. Paired-end sequence was obtained from an Illumina 2500 Hiseq (2 X 101 bp) using the Ribo-Zero rRNA Removal Kit prior to library preparation. Read pairs for each biological replicate were aligned to TAIR10 [43, 44] assembly using Spliced Transcripts Alignment to a Reference (STAR) [45]. Gene level read counts were determined using HTSeq-count. For transcript/isoform analysis, Sailfish (v0.9.0) was used for splice aware transcript quantification [46]. Differential expression was determined using the generalized linear model likelihood test within the Empirical Analysis of Digital Gene Expression Data in R (EdgeR) package [47, 48].

### Protein extraction and data analysis

Soluble and membrane proteins were extracted and processed according to Basu et al. [40]. In brief, seedlings from five plates, were pooled for each of the 3 replicates. Seedlings were crushed in liquid nitrogen and vortexed thoroughly in extraction buffer. Sample were then centrifuged at 100,000 g to isolate the membrane proteins. The supernatant was saved and used for the extraction of soluble proteins. Samples were sent to the Proteomics & Mass Spectrometry Facility at the Donald Danforth Plant Science Center (St. Louis, MO) for processing, iTRAQ labeling, and tandem mass spectrometry of protein samples as detailed in [49, 50]. Briefly, protein concentration of each replicate was estimated using CBX assay. Equal amounts (40 μg) of both membrane and soluble, three replicates each, were digested with trypsin and labeled with a unique iTRAQ 8plex (AB SCIEX, Massachusetts, USA). The labeled replicates were pooled and fractionated using high pH reverse phase chromatography [40, 51]. The fractions were combined into 12 final fractions for LC-MS/MS using an LTQ-Orbitrap Velos Pro mass spectrometer (ThermoFisher Scientific, Waltham, MA).

Mass spectrometry data analyses were automated using Mascot Daemon 2.5 (Matrix Science, London, UK). The TAIR10 database was used as a reference (including target and decoy sequences, 35,386 entries) assuming trypsin digestion. To identify post-translational modifications using Mascot, the modifications due to iTRAQ 8-plex labeling of lysine and the N-terminus of the peptides and carbamidomethyl of cysteine were specified as fixed modifications. The variable modifications were specified as deamidation of asparagine and glutamine, oxidation of methionine, and phosphorylation of serine/threonine and tyrosine. Only proteins with at least two peptides with score > 20 were reported.

A Student’s t-test was used to determine significant difference between protein expression in spaceflight and ground control conditions. In accordance with previous studies demonstrating smaller but impactful variations in protein abundance, proteins with a *p*-value ≤0.05 and a log fold change greater than 0.2 or less than − 0.2 were considered significantly different between experimental and control samples [49, 51]. Individual peptides containing post-translational modifications were examined evaluated using a Student’s t-test to determine phosphorylation events that were altered by spaceflight conditions.

A hypergeometric distribution test was used to determine if overlap between proteomic and transcriptomic candidates was non-random. The hypergeometric *P*-value was calculated using *p = (*_*k*_*C*_*x*_*) (*_*(n-k)*_*C*_*(n-x)*_*) /*_*N*_*C*_*n*_, where N is the number of genes in the genome (estimated at 27,000 for this work), k is the number of genes identified by RNAseq, n is the number of genes identified by proteomics, x is the number of overlapping candidates, and _k_C_x_ is the number of possible gene combinations.

### Exclusions based on control experiments

In addition to the ground control at Kennedy Space Center, two additional analyses were run to provide insight into whether the spaceflight hardware [41] or the preservative RNAlater [42] caused significant changes in the transcriptome and proteome of Arabidopsis seedlings. Comparing differentially expressed genes and proteins from these additional control studies with those from the spaceflight/ground control analysis revealed 263 genes and 159 proteins significantly affected by both hardware and spaceflight while 249 genes and 152 proteins were affected both by RNAlater and spaceflight. Rather than excluding all genes found in these secondary controls, a logical decision rule (Eq. 1) was developed to identify genes which were more or differently perturbed by spaceflight than either control study. All genes and proteins that passed the decision rule were considered differentially expressed despite their altered expression in a control study. The decision rule,

1 $$ \left(\left| Range\left({LFC}_{control},{LFC}_{SF}\right)\right|>\left|{LFC}_{control}\right|\right) or\ \left(\left|{LFC}_{SF}\right|>\left|{LFC}_{control}\right|\right) $$

where *LFC*_*control*_ is the log_2_ fold change of a gene in the control study where it showed significant differential expression and *LFC*_*SF*_ is the log_2_ fold change of a gene in the spaceflight/ground control experiment, allows genes to be retained if the impact of spaceflight was different than the effect of either the preservative or hardware control.

### Subcellular analyses

Differentially expressed transcripts and proteins were analyzed using SUBA4 [13] (V.4.6.0, accessed June 2017) to identify the subcellular localization of differentially expressed genes. Only transcripts with a log-fold change ±1 (FDR ≤ 0.05) or proteins with a log-fold change ±0.2 (*P* ≤ 0.05) were included in the analysis. Multi-level pie charts were rendered to identify the distribution of localization among subsets of genes.

### Gene ontology enrichment analyses

Gene Ontology (GO) enrichments were performed for Biological Processes using Python based tool, Orange [52] (V.3.7.0, GO terms accessed June 2017). Only transcripts with a log-fold change ±1 (FDR ≤ 0.05) or proteins with a log-fold change ±0.2 (P ≤ 0.05) were included in the analysis. The upregulated and downregulated genes were run separately, and the resultant GO enrichments were thinned to include GO terms with a *p*-value ≤0.05 using the Bonferroni correction for multiple comparisons that had at least 4 and no more than 750 reference genes within the term (to exclude poorly annotated terms and overly broad terms, respectively) and that had more than one differentially expressed gene within the experimental dataset. Of the remaining GO terms, only the two terms with minimal distance from biological process within each branch were included. When redundant terms (terms containing identical gene sets and with similar naming) were found, only the most informative GO term was retained. A visual representation of the resulting enrichments was created by analyzing enrichment of associated GO terms and creating a heat map. The heat map was generated using the R packages “RColorBrewer” and “gplots.”

## Supplementary information

**Additional file 1.**

**Additional file 2.**

**Additional file 3.**

## Data Availability

The datasets generated for this study can be found in the NASA GeneLab database at genelab.nasa.gov. They are available as GeneLab Data Set (GLDS)-38 (https://genelab-data.ndc.nasa.gov/genelab/accession/GLDS-38/).

## Abbreviations

BRIC: Biological Research in Canister
EdgeR: Empirical Analysis of Digital Gene Expression Data in R
GO: Gene Ontology
ISS: International Space Station
L2FC: Log2(Fold Change)
PDFUs: Petri Dish Fixation Units
PTMs: Post-translational Modifications
ROS: Reactive Oxygen Species
SAUR: Small Auxin-upregulated RNA
STAR: Spliced Transcripts Alignment to a Reference

## References

[1] Correll MJ, Pyle TP, Millar KDL, Sun Y, Yao J, Edelmann RE (2013). Transcriptome analyses of Arabidopsis thaliana seedlings grown in space: implications for gravity-responsive genes. Planta..

[2] Fengler S, Spirer I, Neef M, Ecke M, Nieselt K, Hampp R (2015). A whole-genome microarray study of Arabidopsis thaliana semisolid callus cultures exposed to microgravity and nonmicrogravity related spaceflight conditions for 5 days on board of Shenzhou 8. Biomed Res Int.

[3] Kwon T, Sparks JA, Nakashima J, Allen SN, Tang Y, Blancaflor EB (2015). Transcriptional response of Arabidopsis seedlings during spaceflight reveals peroxidase and cell wall remodeling genes associated with root hair development. Am J Bot.

[4] Paul A-L, Popp MP, Gurley WB, Guy C, Norwood KL, Ferl RJ (2005). Arabidopsis gene expression patterns are altered during spaceflight. Adv Space Res.

[5] Paul A-L, Amalfitano CE, Ferl RJ (2012). Plant growth strategies are remodeled by spaceflight. BMC Plant Biol.

[6] Paul A-L, Zupanska AK, Schultz ER, Ferl RJ (2013). Organ-specific remodeling of the Arabidopsis transcriptome in response to spaceflight. BMC Plant Biol.

[7] Soh H, Auh C, Soh W-Y, Han K, Kim D, Lee S (2011). Gene expression changes in Arabidopsis seedlings during short- to long-term exposure to 3-D clinorotation. Planta..

[8] Sugimoto M, Oono Y, Gusev O, Matsumoto T, Yazawa T, Levinskikh MA (2014). Genome-wide expression analysis of reactive oxygen species gene network in Mizuna plants grown in long-term spaceflight. BMC Plant Biol.

[9] Ferl RJ, Koh J, Denison F, Paul A-L (2014). Spaceflight induces specific alterations in the proteomes of Arabidopsis. Astrobiology..

[10] Hausmann N, Fengler S, Hennig A, Franz-Wachtel M, Hampp R, Neef M (2014). Cytosolic calcium, hydrogen peroxide and related gene expression and protein modulation in Arabidopsis thaliana cell cultures respond immediately to altered gravitation: parabolic flight data. Plant Biol.

[11] Mazars C, Brière C, Grat S, Pichereaux C, Rossignol M, Pereda-Loth V (2014). Microsome-associated proteome modifications of Arabidopsis seedlings grown on board the international Space Station reveal the possible effect on plants of space stresses other than microgravity. Plant Signal Behav.

[12] Zhang Y, Wang L, Xie J, Zheng H (2015). Differential protein expression profiling of Arabidopsis thaliana callus under microgravity on board the Chinese SZ-8 spacecraft. Planta..

[13] Hooper CM, Castleden IR, Tanz SK, Aryamanesh N, Millar AH (2017). SUBA4: the interactive data analysis centre for Arabidopsis subcellular protein locations. Nucleic Acids Res..

[14] Chiba Y, Oshima K, Arai H, Ishii M, Igarashi Y (2012). Discovery and analysis of cofactor-dependent Phosphoglycerate Mutase homologs as novel Phosphoserine phosphatases in Hydrogenobacter thermophilus. J Biol Chem.

[15] Medina F-J, Herranz R (2010). Microgravity environment uncouples cell growth and cell proliferation in root meristematic cells. Plant Signal Behav.

[16] Streitner C, Köster T, Simpson CG, Shaw P, Danisman S, Brown JWS (2012). An hnRNP-like RNA-binding protein affects alternative splicing by in vivo interaction with transcripts in Arabidopsis thaliana. Nucleic Acids Res.

[17] Berrocal-Lobo M, Stone S, Yang X, Antico J, Callis J, Ramonell KM (2010). ATL9, a RING zinc finger protein with E3 ubiquitin ligase activity implicated in chitin- and NADPH oxidase-mediated defense responses. PLoS One.

[18] Luo Hongli, Laluk Kristin, Lai Zhibing, Veronese Paola, Song Fengming, Mengiste Tesfaye (2010). The Arabidopsis Botrytis Susceptible1 Interactor Defines a Subclass of RING E3 Ligases That Regulate Pathogen and Stress Responses. Plant Physiology.

[19] Li H, Jiang H, Bu Q, Zhao Q, Sun J, Xie Q (2011). The Arabidopsis RING finger E3 ligase RHA2b acts additively with RHA2a in regulating abscisic acid signaling and drought response. Plant Physiol.

[20] Hoson T, Soga K, Wakabayashi K, Kamisaka S, Tanimoto E (2003). Growth and cell wall changes in rice roots during spaceflight. Plant Soil.

[21] Nedukha EM (1997). Effects of microgravity on the structure and function of plant cell walls. Int Rev Cytol.

[22] Spartz AK, Ren H, Park MY, Grandt KN, Lee SH, Murphy AS (2014). SAUR inhibition of PP2C-D phosphatases activates plasma membrane H+-ATPases to promote cell expansion in Arabidopsis. Plant Cell.

[23] Ban Y, Kobayashi Y, Hara T, Hamada T, Hashimoto T, Takeda S (2013). α-Tubulin is rapidly phosphorylated in response to hyperosmotic stress in rice and Arabidopsis. Plant Cell Physiol.

[24] Stortenbeker N, Bemer M (2019). The SAUR gene family: the plant’s toolbox for adaptation of growth and development. J Exp Bot.

[25] Paul A-L, Sng NJ, Zupanska AK, Krishnamurthy A, Schultz ER, Ferl RJ (2017). Genetic dissection of the Arabidopsis spaceflight transcriptome: are some responses dispensable for the physiological adaptation of plants to spaceflight?. PLoS One.

[26] Dietz K-J, Turkan I, Krieger-Liszkay A (2016). Redox- and reactive oxygen species-dependent signaling into and out of the photosynthesizing chloroplast. Plant Physiol.

[27] Gilroy S, Białasek M, Suzuki N, Górecka M, Devireddy AR, Karpiński S (2016). ROS, calcium, and electric signals: key mediators of rapid systemic signaling in plants. Plant Physiol.

[28] Fu X, Fu N, Guo S, Yan Z, Xu Y, Hu H (2009). Estimating accuracy of RNA-Seq and microarrays with proteomics. BMC Genomics.

[29] Taniguchi Y, Choi PJ, Li G-W, Chen H, Babu M, Hearn J (2010). Quantifying E. coli proteome and Transcriptome with single-molecule sensitivity in single cells. Science..

[30] Ghazalpour A, Bennett B, Petyuk VA, Orozco L, Hagopian R, Mungrue IN (2011). Comparative analysis of proteome and Transcriptome variation in mouse. PLoS Genet.

[31] Bai Y, Wang S, Zhong H, Yang Q, Zhang F, Zhuang Z (2015). Integrative analyses reveal transcriptome-proteome correlation in biological pathways and secondary metabolism clusters in A flavus in response to temperature. Sci Rep.

[32] Choudhury FK, Rivero RM, Blumwald E, Mittler R (2017). Reactive oxygen species, abiotic stress and stress combination. Plant J.

[33] Li J, Henty-Ridilla JL, Huang S, Wang X, Blanchoin L, Staiger CJ (2012). Capping protein modulates the dynamic behavior of actin filaments in response to phosphatidic acid in Arabidopsis. Plant Cell.

[34] Li J, Henty-Ridilla JL, Staiger BH, Day B, Staiger CJ (2015). Capping protein integrates multiple MAMP signalling pathways to modulate actin dynamics during plant innate immunity. Nat Commun.

[35] Li J, Cao L, Staiger CJ (2017). Capping protein modulates actin remodeling in response to reactive oxygen species during plant innate immunity. Plant Physiol.

[36] Livanos P, Galatis B, Apostolakos P. The interplay between ROS and tubulin cytoskeleton in plants. Plant Signal Behav. 2014 [cited 2019 Jul 30];9. Available from: https://www.ncbi.nlm.nih.gov/pmc/articles/PMC4091245/. 10.4161/psb.28069 PMC4091245 24521945

[37] Leyser O (2018). Auxin Signaling. Plant Physiol.

[38] Haruta M, Sabat G, Stecker K, Minkoff BB, Sussman MR (2014). A peptide hormone and its receptor protein kinase regulate plant cell expansion. Science..

[39] Polko JK, Barnes WJ, Voiniciuc C, Doctor S, Steinwand B, Hill JL (2018). SHOU4 Proteins Regulate Trafficking of Cellulose Synthase Complexes to the Plasma Membrane. Curr Biol..

[40] Jones DM, Murray CM, Ketelaar KJ, Thomas JJ, Villalobos JA, Wallace IS. The Emerging Role of Protein Phosphorylation as a Critical Regulatory Mechanism Controlling Cellulose Biosynthesis. Front Plant Sci. 2016 [cited 2019 Sep 25];7. Available from: https://www.ncbi.nlm.nih.gov/pmc/articles/PMC4877384/. 10.3389/fpls.2016.00684 PMC4877384 27252710

[41] Basu P, Kruse CPS, Luesse DR, Wyatt SE (2017). Growth in spaceflight hardware results in alterations to the transcriptome and proteome. Life Sci Space Res.

[42] Kruse CPS, Basu P, Luesse DR, Wyatt SE (2017). Transcriptome and proteome responses in RNAlater preserved tissue of Arabidopsis thaliana. PLoS One.

[43] Lamesch P, Berardini TZ, Li D, Swarbreck D, Wilks C, Sasidharan R (2012). The Arabidopsis information resource (TAIR): improved gene annotation and new tools. Nucleic Acids Res.

[44] Swarbreck D, Wilks C, Lamesch P, Berardini TZ, Garcia-Hernandez M, Foerster H (2008). The Arabidopsis information resource (TAIR): gene structure and function annotation. Nucleic Acids Res.

[45] Dobin A, Davis CA, Schlesinger F, Drenkow J, Zaleski C, Jha S (2013). STAR: ultrafast universal RNA-seq aligner. Bioinformatics..

[46] Patro R, Mount SM, Kingsford C (2014). Sailfish enables alignment-free isoform quantification from RNA-seq reads using lightweight algorithms. Nat Biotechnol.

[47] Robinson MD, McCarthy DJ, Smyth GK (2010). edgeR: a bioconductor package for differential expression analysis of digital gene expression data. Bioinformatics..

[48] Nikolayeva O, Robinson MD (2014). edgeR for differential RNA-seq and ChIP-seq analysis: an application to stem cell biology. Methods Mol Biol.

[49] Alvarez S, Roy Choudhury S, Pandey S (2014). Comparative quantitative proteomics analysis of the ABA response of roots of drought-sensitive and drought-tolerant wheat varieties identifies proteomic signatures of drought adaptability. J Proteome Res.

[50] Alvarez S, Roy Choudhury S, Sivagnanam K, Hicks LM, Pandey S (2015). Quantitative proteomics analysis of Camelina sativa seeds overexpressing the AGG3 gene to identify the proteomic basis of increased yield and stress tolerance. J Proteome Res.

[51] Yang F, Shen Y, Camp DG, Smith RD (2012). High-pH reversed-phase chromatography with fraction concatenation for 2D proteomic analysis. Expert Rev Proteomics.

[52] Demšar J, Curk T, Erjavec A, Gorup Č, Hočevar T, Milutinovič M (2013). Orange: data mining toolbox in Python. J Mach Learn Res.

