# Supplementary material for: Spaceflight induces novel regulatory responses in Arabidopsis seedling as revealed by combined proteomic and transcriptomic analyses
Source: BMC Plant Biol. 2020 May 27;20:237. doi: 10.1186/s12870-020-02392-6 (PMC7251690; doi:10.1186/s12870-020-02392-6)
Supplement: Supplementary file 3 — Additional file 3. [file 12870_2020_2392_MOESM3_ESM.docx]

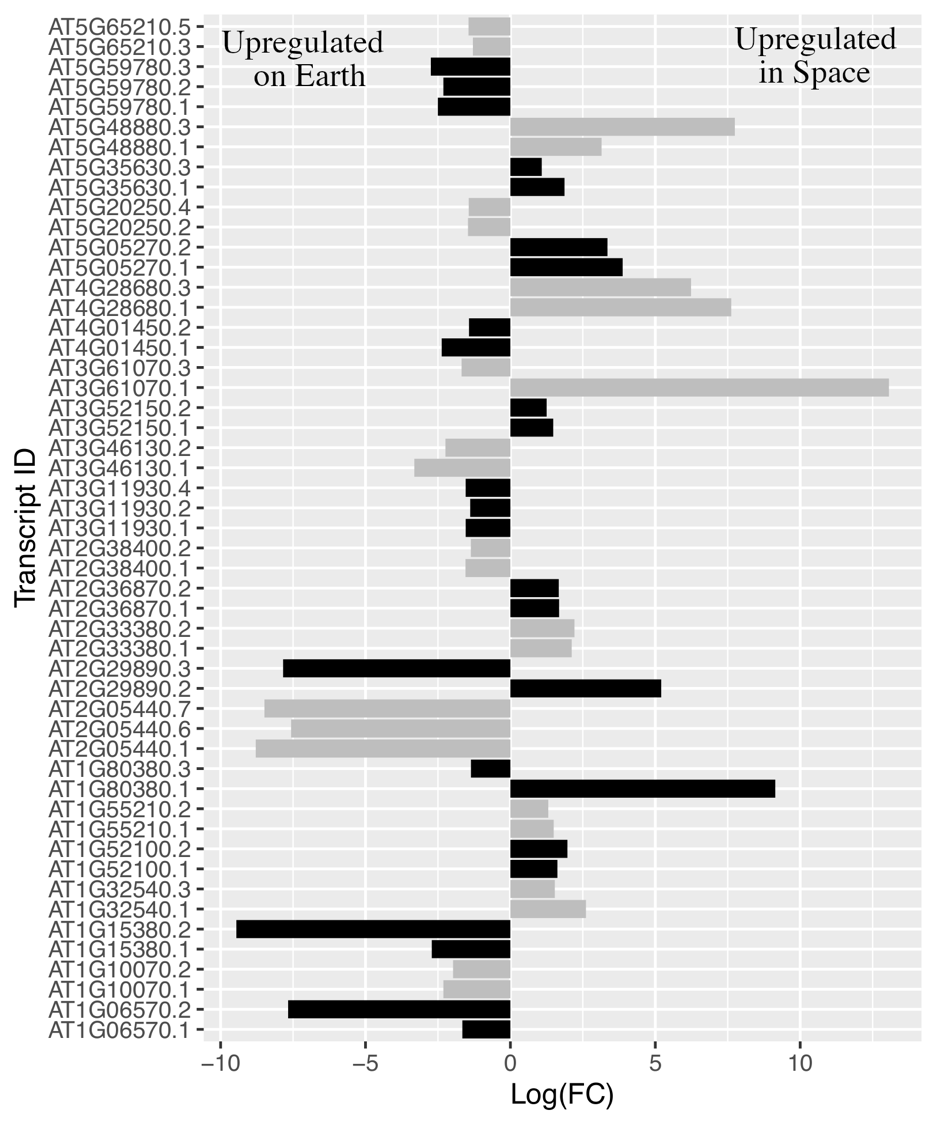


**Fig S1.** Log_2_ fold change in expression is plotted for all isoforms where >2 isoforms were found to be differentially expressed. Bar coloring alternates to delineate isoform groups belonging to a shared gene.
